# Regio- and stereochemical stability induced by anomeric and *gauche* effects in difluorinated pyrrolidines

**DOI:** 10.3762/bjoc.20.140

**Published:** 2024-07-12

**Authors:** Ana Flávia Candida Silva, Francisco A Martins, Matheus P Freitas

**Affiliations:** 1 Department of Chemistry, Institute of Natural Sciences, Federal University of Lavras, 37200-900, Lavras, MG, Brazil; 2 Department of Chemistry, University of Houston, Houston, TX, USA

**Keywords:** anomeric effect, fluoropyrrolidine, *gauche* effect, stereochemistry

## Abstract

Selective fluorination of the pyrrolidine ring in proline motifs has been found to induce significant conformational changes that impact the structure and biological roles of modified peptides and proteins. Vicinal difluorination of fluoroproline, for example, in (3*S*,4*R*)-3,4-difluoroproline, serves to mitigate the inherent conformational bias of the pyrrolidine ring by inducing stereoelectronic effects that attenuate this conformational bias. In this investigation, we present a quantumchemical analysis of the conformational equilibrium and effects that are induced in difluorinated pyrrolidines, with a particular focus on exploring the impact of *gauche* and anomeric effects on the conformer stabilities of different stereo- and regioisomers. Initially, we conducted a benchmark assessment comparing the optimal density functional theory method with coupled cluster with single and double excitations (CCSD) calculations and crystallographic data using the 3-fluoropyrrolidinium cation and 3-fluoropyrrolidine. Subsequently, we explored the relative energy of all favored conformations of all different stereoisomers of 2,3-, 2,4-, and 3,4-difluoropyrrolidines at the B3LYP-D3BJ/6-311++G** level. A generalized anomeric effect, arising from n_N_→σ*_CF_ electron delocalization, is particularly important in modulating the energetics of the α-fluoro isomers and imparts a strong conformational bias. In contrast, the fluorine *gauche* effect assumes a secondary role, as it is overshadowed by steric and electrostatic interactions, referred to as Lewis interactions from a natural bond orbital perspective.

## Introduction

The pyrrolidine ring structure is prevalent in numerous natural alkaloids and is an important feature of the proline and hydroxyproline residues that pervade biochemistry in peptides and proteins. The chemical and biological properties of substituted pyrrolidine derivatives, along with many other compounds, hinge on the relative stereochemistry. It is well established that the presence of fluorine in an organic molecule can significantly influence the stereochemical behavior. Consequently, various molecular properties, such as polarity, viscosity, and intra- and intermolecular interactions, are impacted by the C‒F bond. These features underlie the important role of selective fluorination in pharmaceuticals and agrochemicals development [1].

For instance, substituted 3-fluoropyrrolidines, particularly in the form of 2-carboxy derivatives (fluoroprolines), have been extensively explored. These compounds represent valuable nonnatural amino acids, and depending on the regio- and stereochemistry of fluorine substitution, fluoroproline substututions can enhance the conformational stability of proline-rich proteins such as collagen [2]. Therefore, pyrrolidine derivatives are particularly susceptible to conformational control induced by a fluorine substituent.

**Figure 1:**
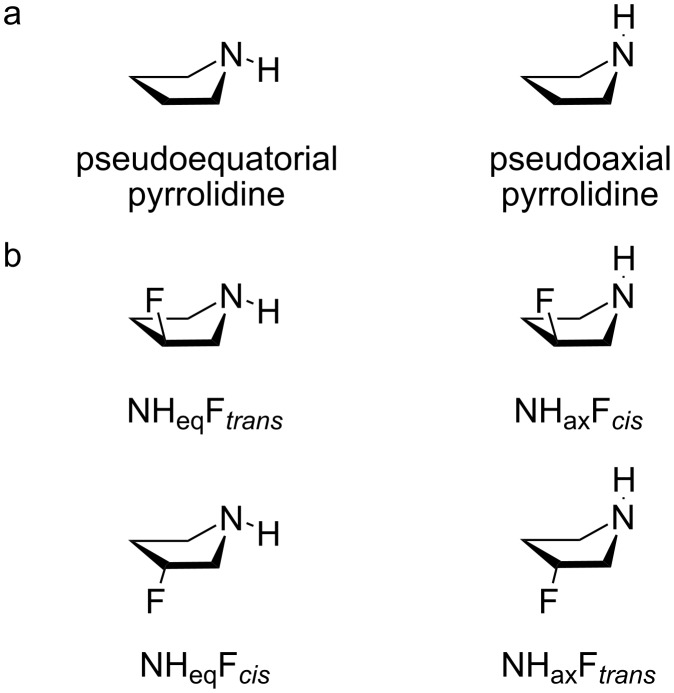
a) Pseudoequatorial and pseudoaxial conformations of pyrrolidine. b) *Cis*- and *trans*-isomers of 3-fluoropyrrolidine.

The 5-membered pyrrolidine ring is a cyclic alkylamine that adopts a conformation that resembles the familiar envelope of cyclopentane, with an NH unit occupying either a pseudoaxial or pseudoequatorial position (Figure 1a). When a hydrogen atom of the pyrrolidine at C-3 is replaced with fluorine, a conformational interconversion can occur within the *cis*- and *trans*-isomers, as illustrated in Figure 1b. The fluorine *gauche* effect, commonly observed in compounds containing the F‒C‒C‒X fragment (where X is an electron-withdrawing substituent, such as nitrogen), typically favors a conformation where F and X are *gauche* to each other. This preference is attributed to stabilizing σ_CH_→σ*_CF_ and σ_CH_→σ*_CX_ hyperconjugative interactions [3]. However, it is noteworthy that 3-fluoropiperidine does not exhibit such a fluorine *gauche* effect. In this case, the axial conformer is similarly populated to the equatorial conformer, despite the favorable antiperiplanar arrangement of orbitals that would facilitate these interactions [4].

Protonation of 3-fluoropyrrolidine generates the 3-fluoropyrrolidinium cation, and this typically results in a highly favored conformation in both the gas phase and solution where the fluorine and nitrogen atoms are *cis*, mirroring the behavior observed in analagous 4- and 6-membered ring systems [5]. This conformational preference is attributed to an electrostatic *gauche* effect, where an attractive NH_2_^+^∙∙∙F^δ−^ interaction reinforces the well-known hyperconjugative *gauche* effect. Additionally, NH∙∙∙F hydrogen bonding has been proposed to play a role in stabilizing conformers of certain 3-fluoroalkylamines and the respective cations [6].

Intramolecular hydrogen bonds involving either the carboxy or hydroxy group of 4*R*- and 4*S*-hydroxyproline have been identified as key factors in stabilizing the favored conformations in the gas phase. Therein, the contribution of a *gauche* effect due to electron delocalization is considered to be secondary [7]. However, stabilization via intramolecular hydrogen bonding does not seem to significantly impact the conformational stability of 3-fluoropiperidine. In this context, the *cis*-conformer, with the axial fluorine atom facing the *N*-hydrogen atom, is either equally or only slightly more stable than the other three conformers in both the gas phase and implicit water [4]. Hence, it appears that strong intramolecular interactions, and not only hydrogen bonding, govern the orientation of the fluorine atom in the F‒C‒C‒N fragment, favoring the *cis*-isomer of 3-fluoropyrrolidine.

**Figure 2:**
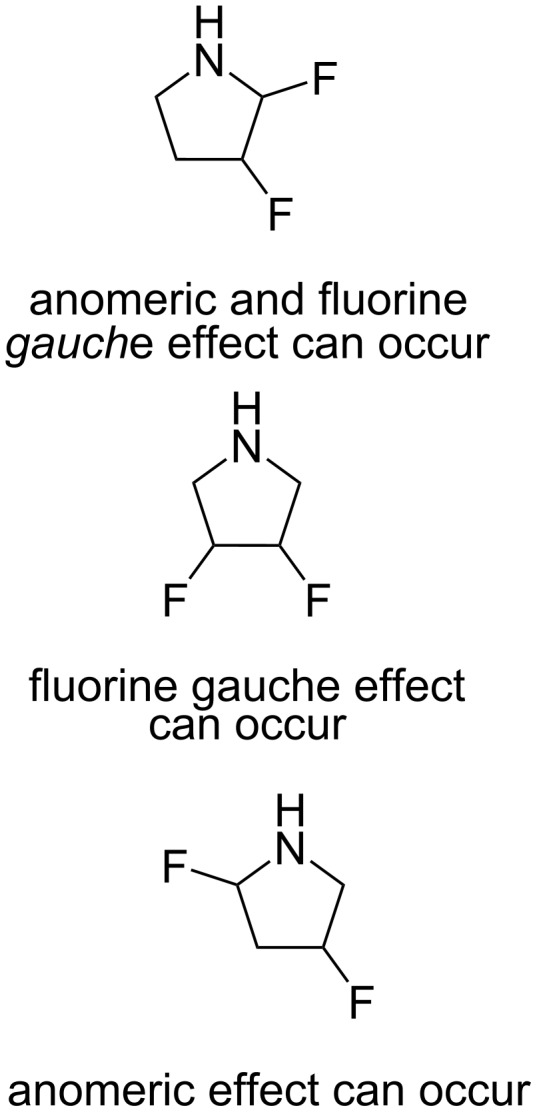
Flat representations of 2,3-, 3,4-, and 2,4-difluoropyrrolidines. The potential effects resulting from the addition of a second 1,2- or 1,3-fluorine atom (n_N_→σ*_CF_ anomeric and σ_CH_→σ*_CF_ fluorine *gauche* effects) on the conformation of different stereoisomers is explored.

To investigate potential changes in the preferred orientation of the fluorine substituent in 3-fluoropyrrolidine and the respective cation, an additional fluorine atom was introduced into the molecule for theoretic studies (Figure 2). The subsequent evaluation focused on the role of anomeric and fluorine *gauche* effects to evaluate significant conformational biases in all of the possible isomers.

Achieving conformational control upon the introduction of a second fluorine atom in fluoropyrrolidine presents challenges. Unlike the chair-like conformation of six-membered rings, five-membered rings lack the geometric arrangement necessary to most effectively accommodate anomeric and *gauche* effects [8–9]. It is noteworthy that vicinal difluorination has previously been demonstrated to minimize conformational bias in fluoroprolines [10], offering some insight into the potential outcomes of this study.

In this study, initially, the conformational equilibrium of 3-fluoropyrrolidine and the corresponding cation were analyzed to establish a benchmark for selecting an appropriate theory level for subsequent calculations. Then, the different isomers of the 1,2- or 1,3-difluorinated pyrrolidines were each be subjected to quantum-chemical analysis.

## Results and Discussion

In order to achieve the most accurate conformational depiction of the difluorinated pyrrolidines through density functional theory (DFT), a benchmark study was conducted. This study compares the crystallographic geometry of a pyrrolidinium salt [11] with the CCSD/DGTZVP geometry of the pyrrolidinium cation (with an exclusively axial C–F bond) and the energy of 3-fluoropyrrolidine conformations. Various combinations of DFT methods (B3LYP-D3BJ, ωB97XD, and PBEPBE) and basis sets (DGTZVP and 6-311++G**) were evaluated. The selection of these functionals was based on whether or not they included dispersion terms that could influence the absolute energy. These functionals are widely used and have demonstrated strong performance in numerous evaluations and validations across the literature. Similarly, for the basis sets, a comparison was made between a triple-zeta valence plus polarization basis set with diffuse and tight d-functions (DGTZVP) and a Pople-based large basis set with polarizability and diffuse functions, often referred to as high standard.

**Figure 3:**
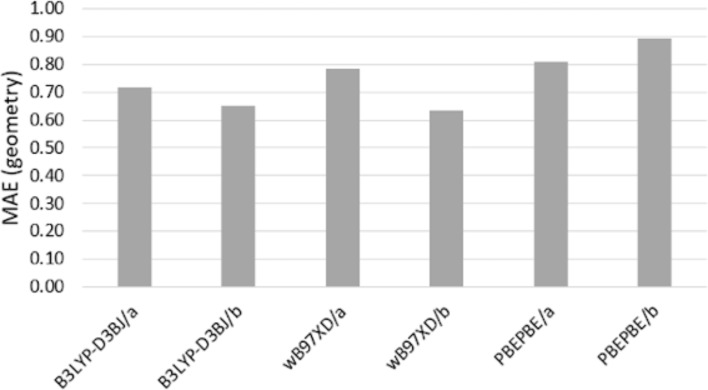
MAE comparing the geometry parameters (bond length, bond angle, and dihedral angle) obtained from DFT calculations at the CCSD/DGTZVP level. The MAE for the crystal structure compared to the CCSD structure is 20.3316. “a” denotes DGTZVP and “b” denotes 6-311++G**.

The geometry was assessed based on atom distance, bond length, dihedral angle, etc. The analysis revealed that the lowest mean absolute error (MAE) [12] was observed for the B3LYP-D3BJ/6-311++G** and ωB97XD/6-311++G** levels (Figure 3). While there were differences between the CCSD and experimental structures, attributed to intermolecular forces and counterion effects, these differences did not impact the selection of the best levels based on geometry criteria.

Regarding the conformational energy, 3-fluoropyrrolidine exhibited four conformers in the gas phase, although this was reduced to three in implicit DMSO experiments (Table 1). In both cases, a *cis*-twist ring with an axial N–H bond was the most stable conformer, allowing for an intramolecular F∙∙∙H hydrogen bond. However, 3-fluoropyrrolidine displayed extensive conformational diversity (Δ*G*^0^ among conformers ≤1.6 kcal⋅mol^−1^) as the intramolecular interactions were not sufficiently stabilizing to dictate a dominant conformation. Consequently the introduction of a second fluorine atom with an appropriate relative configuration was thought to reinforce and establish a predominant conformation.

**Table 1:** Conformational energy (Δ*G*^0^, kcal⋅mol^−1^) and population (%) of 3-fluoropyrrolidine according to different theoretical levels for the gas phase and implicit DMSO (in parentheses), along with the MAE compared to the CCSD/DGTZVP level.

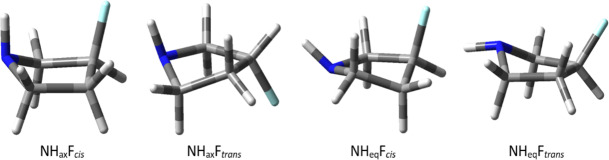								

conformer	CCSD/a^a^	B3LYP-D3BJ/a	B3LYP-D3BJ/b^b^	ωB97XD/a	ωB97XD/b	PBEPBE/a	PBEPBE/b	average, %

NH_ax_F*_cis_*	0.00 (0.00)	0.00 (0.00)	0.00 (0.00)	0.00 (0.00)	0.00 (0.00)	0.00 (0.00)	0.00 (0.00)	54 (64)
NH_ax_F*_trans_*	1.00 (0.46)	0.97 (0.47)	0.94 (0.47)	0.96 (0.49)	0.91 (0.47)	0.97 (0.51)	0.96 (0.53)	11 (28)
NH_eq_F*_cis_*	0.28 (—)	0.31 (—)	0.28 (—)	0.20 (—)	0.09 (—)	0.61 (—)	0.56 (—)	31 (—)
NH_eq_F*_trans_*	1.60 (1.17)	1.59 (1.27)	1.45 (1.09)	1.57 (1.21)	1.49 (1.07)	1.64 (1.35)	1.46 (1.15)	4 (9)
MAE	—	0.02 (0.04)	0.05 (0.03)	0.04 (0.02)	0.10 (0.04)	0.10 (0.08)	0.12 (0.03)	—

^a^a = DGTZVP. ^b^b = 6-311++G**.

**Figure 4:**
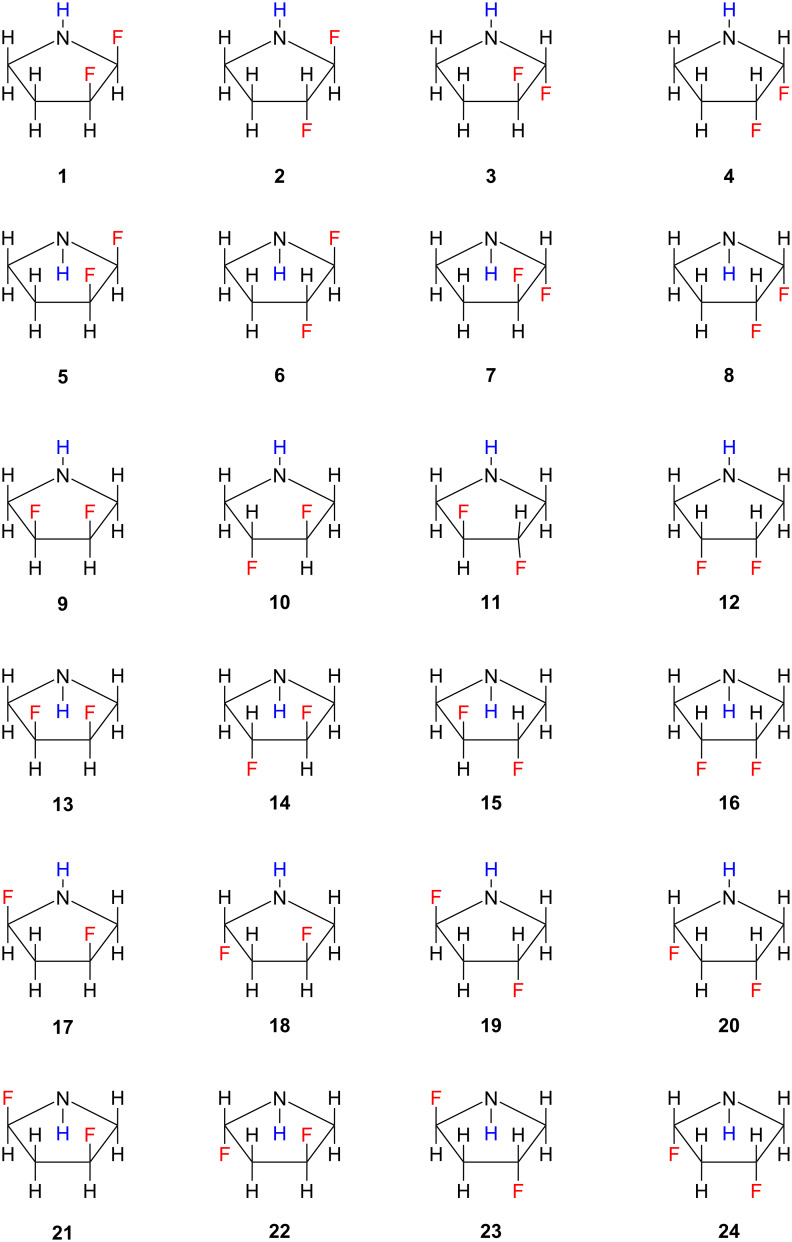
Exhaustive illustration of all conformational, configurational, and constitutional isomers of difluorinated pyrrolidine, illustrating the relative orientation of the C–F and N–H bonds used to build the input geometry for the calculations. The pairs **10**/**11** and **14**/**15** were degenerate. Consequently, only one structure of each pair was computed.

Theoretical levels that closely approximated the CCSD outcome included B3LYP-D3BJ/6-311++G**, B3LYP-D3BJ/DGTZVP, and ωB97XD/DGTZVP (Table 1). Consequently, the B3LYP-D3BJ/6-311++G** level was selected for further calculations involving the difluorinated pyrrolidines shown in Figure 4.

From the pool of 24 potential conformational, configurational, and constitutional isomers of the difluorinated pyrrolidines (i.e., **1**–**24**), two pairs were degenerate (i.e., **10**/**11** and **14**/**15**), reducing the number of distinct structures to 22. However, due to the considerable flexibility of the five-membered ring and the low barrier for pyramidal interconversion of the N–H moiety, some inputs converged to the same isomer post-geometry optimization. Consequently, a total of 10 different structures was identified in the gas phase, with two additional structures observed in implicit DMSO (using solvation model density, SMD), bringing the total to 12.

**Figure 5:**
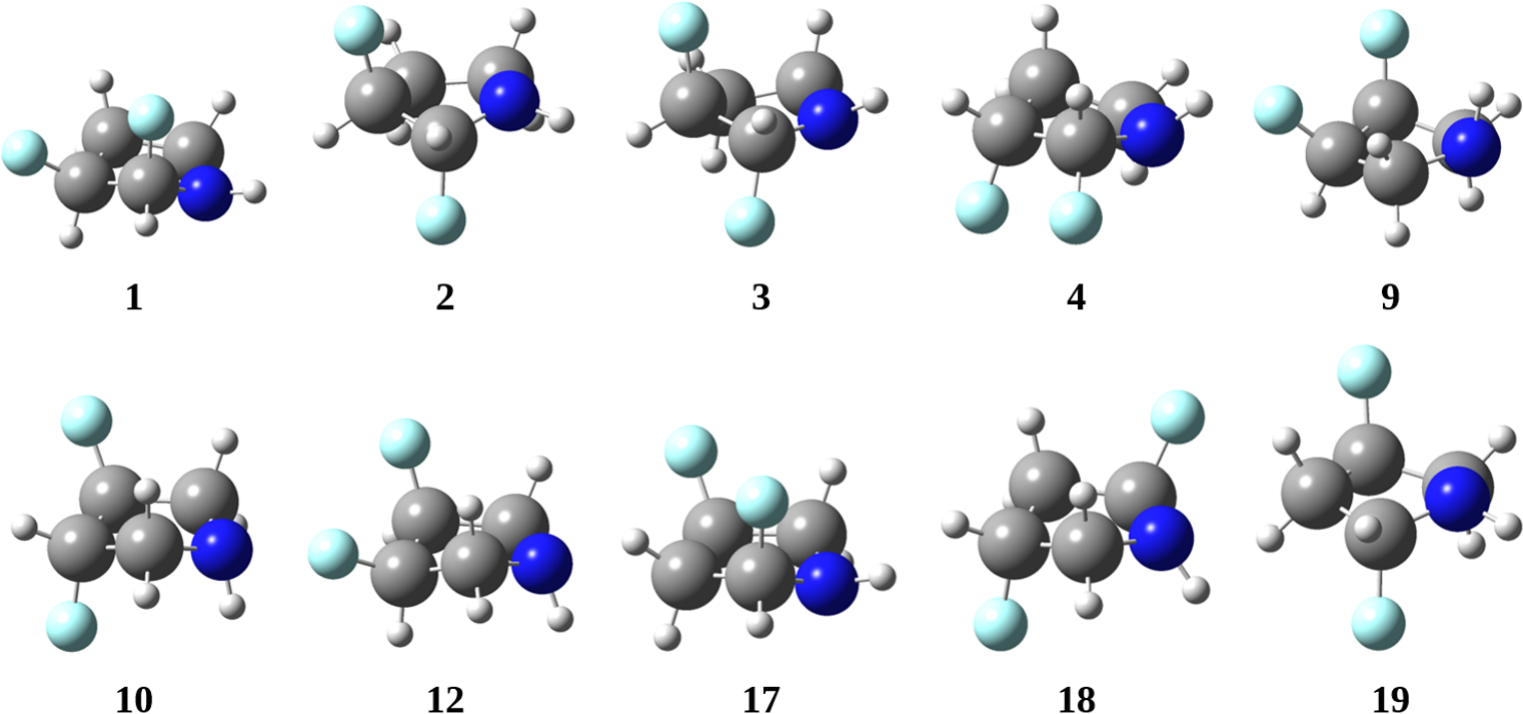
Stable difluorinated pyrrolidines derived from gas-phase calculations performed at the B3LYP-D3BJ/6-311++G** level. Conformers: **1**/**4**, **2**/**3**, **9**/**12**, and **18**/**19**. Stereoisomers: **1** /**4**, **9**/**12**, and **17**/**19**.

Since the atomic composition of the compounds in Figure 4 was the same, the relative energy may be compared, and a stability landscape covering all optimized structures may be obtained from this analysis. Among these structures, only six were found to be significantly stable (<3 kcal⋅mol^−1^) in either the gas phase or DMSO. Remarkably, structure **19** was the most stable, followed by structure **17**, as shown in Figure 5 and Table 2. This disparity in stability was largely attributed to the relative orientation of the nitrogen electron lone pair and the adjacent C–F bond, facilitating an anomeric interaction characterized by n_N_→σ*_CF_ electron delocalization. Isomer **2** also demonstrated such an orientation, but the C–F bonds in this case were vicinal. Compounds lacking α-fluorine atoms (i.e., **9**, **10**, **12**, and **16**) possessed a notably higher energy, indicating the impact of the anomeric effect.

**Table 2:** Relative Gibbs free energy (kcal⋅mol^−1^) for the geometry-optimized difluorinated pyrrolidines.

isomer	Δ*G*^0^_gas_	isomer	Δ*G*^0^_DMSO_

**1**	3.50	**1**	3.23
**2**	1.15	**2**	1.83
**3**	1.79	**3**	2.20
**4**	7.59	**4**	3.23
**9**	13.02	**5**	3.47
**10**	9.70	**6**	2.19
**12**	13.55	**9**	12.94
**17**	0.75	**10**	10.88
**18**	1.79	**12**	12.87
**19**	0.00	**17**	0.11
		**18**	1.01
		**19**	0.00

**Figure 6:**
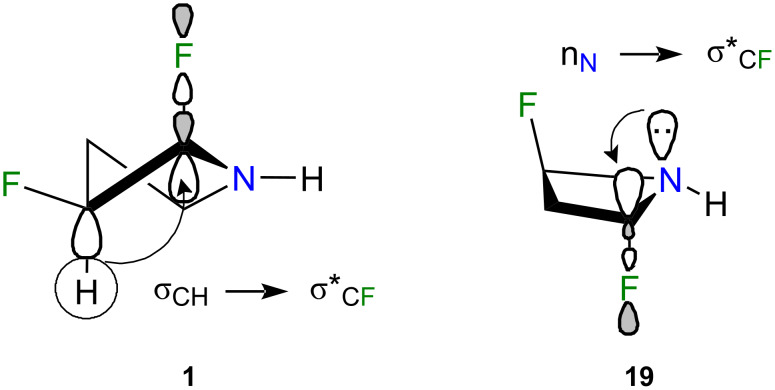
σ_CH_→σ*_CF_ fluorine *gauche* interaction, which also occurred in **19**, and anomeric interaction in isomer **19**. The n_N_→σ*_CF_ electron delocalization stabilized **19** by 22.43 kcal⋅mol^−1^.

To further illuminate these findings, a natural bond orbital (NBO) analysis was conducted across the entire array of difluorinated pyrrolidines to assess the relative energy in isodesmic relationships (Table 3). Unlike in 1,2-difluoroethane [10,13], vicinal *gauche*-oriented fluorine atoms in 5-membered rings, particularly in compounds **1**, **9**, and **12**, as well as in 6-membered rings, do not favor σ_CH_→σ*_CF_ interactions. Rather, less stabilizing σ_CC_→σ*_CF_ interactions were anticipated. Moreover, besides the anomeric interaction, the pseudoaxially oriented C–F bonds in **17** and **19** facilitated efficient electron donation from vicinal antiperiplanar C–H bonds through σ_CH_→σ*_CF_ interactions (Figure 6). The methylene group separating both C–F bonds in **17** and **19** enabled an additional σ_CH_→σ*_CF_ interaction, rather than the negligible σ_CF_→σ*_CF_ electron delocalization that would occur if these bonds were vicinally antiperiplanar. As discerned from the decomposition of the full electronic energy into Lewis (e.g., steric effects) and non-Lewis (electron delocalization) contributions, **17** and **19** were favored by a delicate equilibrium between weak repulsion and substantial stabilization due to hyperconjugation. Conversely, structures such as **9** and **10** experienced minimal steric effects but were inadequately stabilized by electron delocalization, rendering them higher in energy when compared to the other isomers. Compounds **1** and **4**, with *syn*-C–F bonds, exhibited the highest Δ*E*_Lewis_ term due to significant dipolar and steric repulsions.

**Table 3:** Relative electronic energy (Δ*E*_full_ = Δ*E*_Lewis_ + Δ*E*_non-Lewis_) in isodesmic relationships and significant electron delocalization derived from NBO calculations (kcal⋅mol^−1^) for the difluorinated pyrrolidines in the gas phase.

isomer	Δ*E*_full_	Δ*E*_Lewis_	Δ*E*_non-Lewis_	n_N_→σ*_CF_	σ_CH_→σ*_CF_^a^

**1**	3.27	31.68	−28.41	23.71	3.81
**2**	1.38	28.39	−27.01	26.97	3.82
**3**	1.91	27.09	−25.18	22.90	4.61
**4**	7.69	30.35	−22.66	17.53	4.36
**9**	13.09	16.87	−3.79	0.00	8.95
**10**	9.63	9.63	0.00	0.00	5.83
**12**	13.45	20.12	−6.67	1.51	6.37
**17**	1.00	23.20	−22.21	21.97	7.89
**18**	2.07	21.97	−19.90	17.31	8.37
**19**	0.00	22.71	−22.71	22.43	8.02

^a^Sum of antiperiplanar σ_CH_→σ*_CF_ interactions.

## Conclusion

Quantum-chemical calculations were conducted at the B3LYP-GD3BJ/6-311++G(d,p) level to assess isodesmic relationships for the most stable conformations of all isomers of 1,2- and 1,3- difluorinated pyrrolidines. This level of theory was chosen for its superior performance compared to other methodologies, as demonstrated through comparisons of the method with structural data on the 3-fluoropyrrolidinium cation and 3-fluoropyrrolidine. The conformational space of 2,3-, 2,4-, and 3,4-difluoropyrrolidines is notably dictated, both in the gas phase and implicit polar solution, by the N to C–F bond anomeric effect. Additionally, albeit less significant, electron delocalization from the C–H bonding orbital to the C–F antibonding orbital plays a crucial role in lowering the energy of isomers **17** and **19** relative to **1**, **2**, and **3**. These insights deepen our understanding of the energetic principles governing molecular structures and provide valuable guidance in designing selectively fluorinated stereoisomers to influence preferred conformations when designing functional molecules.

## Experimental

### Computational details

The benchmark study was conducted for 3-fluoropyrrolidine and 3-fluoropyrrolidinium cation to determine the optimal theoretical level for further investigations involving 2,3-, 2,4-, and 3,4-difluoropyrrolidines, compared to the CCSD/DGTZVP level [14–15]. The evaluated levels encompassed the B3LYP-GD3BJ [16–18], ωB97XD [19], and PBEPBE [20] functionals, along with the 6-311++G(d,p) [21] and DGTZVP [15] basis sets. Following the identification of B3LYP-GD3BJ/6-311++G(d,p) as the most reliable method, it was employed to optimize the geometry and compute frequencies (to derive Gibbs free energy) for the difluorinated pyrrolidines. These computations were conducted in both the gas phase and employing an implicit DMSO solvent using SMD [22]. Subsequently, a NBO [23] analysis was performed for the gas-phase-optimized geometry at the same level of theory, utilizing the DEL (NOSTAR) keyword to discern Lewis- and non-Lewis-type contributions to the total electronic energy, alongside individual electronic delocalization interactions. The calculations were all performed using the Gaussian 16 package of software [24].

## Supporting Information

File 1 Standard orientations for the difluorinated pyrrolidines in the gas phase and DMSO.

## Data Availability

All data that supports the findings of this study is available in the published article and/or the supporting information to this article.
