# Supplementary material for: Regio- and stereochemical stability induced by anomeric and gauche effects in difluorinated pyrrolidines
Source: Beilstein J Org Chem. 2024 Jul 12;20:1572–9. doi: 10.3762/bjoc.20.140 (PMC11250193; doi:10.3762/bjoc.20.140)
Supplement: File 1 — Standard orientations for the difluorinated pyrrolidines in the gas phase and DMSO. [file Beilstein_J_Org_Chem-20-1572-s001.pdf]

BEILSTEIN JOURNAL OF ORGANIC CHEMISTRY

Supporting Information

for

Regio- and stereochemical stability induced by anomeric and *gauche* effects in difluorinated pyrrolidines

Ana Flávia Candida Silva, Francisco A. Martins and Matheus P. Freitas

Beilstein J. Org. Chem. **2024,** *20,* 1572–1579. doi:10.3762/bjoc.20.140

Standard orientations for the difluorinated pyrrolidines in the gas phase and DMSO

License and Terms: This is a supporting information file under the terms of the Creative Commons Attribution License (https://creativecommons.org/licenses/by/4.0). Please note that the reuse, redistribution and reproduction in particular requires that the author(s) and source are credited and that individual graphics may be subject to special legal provisions.
The license is subject to the *Beilstein Journal of Organic Chemistry* terms and conditions: (https://www.beilstein-journals.org/bjoc/terms)

Pages S2–S6: Standard orientations for the difluorinated pyrrolidines (gas phase, B3LYP-GD3BJ/6-311++G**).

Pages S7–S12: Standard orientations for the difluorinated pyrrolidines (DMSO, B3LYP-GD3BJ/6-311++G**).

Standard orientations for the difluorinated pyrrolidines (gas phase, B3LYP-GD3BJ/6-311++G**)

1

			Coordinates (Angstroms)		
Center Number	Atomic Number	Atomic Type	X	Y	Z
1	6	0	-0.207852	-1.337734	-0.400837
2	6	0	0.697527	-0.489806	0.495450
3	6	0	0.016870	0.879619	0.467467
4	6	0	-1.606124	-0.700101	-0.229161
5	1	0	0.146700	-1.232763	-1.427113
6	1	0	-0.186703	-2.393517	-0.133551
7	1	0	0.730413	-0.852481	1.525352
8	1	0	0.330855	1.582063	1.238604
9	1	0	-2.285022	-1.346602	0.331479
10	1	0	-2.064415	-0.485346	-1.199220
11	7	0	-1.337871	0.528173	0.547253
12	9	0	0.358288	1.517676	-0.771125
13	9	0	2.007517	-0.447063	0.035434
14	1	0	-2.001499	1.284043	0.457379
G° (Hartree-Fock)	-411.1320928		Imag. Freq. =	0	

2

			Coordinates (Angstroms)		
Center Number	Atomic Number	Atomic Type	X	Y	Z
1	6	0	-0.726367	-0.829764	0.900838
2	6	0	-0.710186	0.599955	0.363490
3	6	0	0.608253	0.670790	-0.399903
4	6	0	0.179231	-1.635418	-0.061812
5	1	0	-0.316849	-0.836240	1.911860
6	1	0	-1.748224	-1.206089	0.942732
7	1	0	-0.793146	1.373293	1.125034
8	1	0	0.689997	1.476620	-1.126895
9	1	0	-0.385474	-2.358069	-0.654301
10	1	0	0.951067	-2.177647	0.4933355
11	7	0	0.746107	-0.604317	-0.943427
12	1	0	1.599440	-0.813595	-1.439387
13	9	0	1.617503	0.975409	0.603751
14	9	0	-1.764630	0.795544	-0.544205
G° (Hartree-Fock)	-411.1358488		Imag. Freq. =	0	

3

			Coordinates (Angstroms)		
Center Number	Atomic Number	Atomic Type	X	Y	Z
1	6	0	0.971306	-0.390336	0.983068
2	6	0	0.362726	0.845371	0.340660

3	6	0	-0.850108	0.289100	-0.405349
4	6	0	0.714522	-1.491054	-0.059176
5	1	0	2.026826	-0.249700	1.214486
6	1	0	0.432212	-0.603765	1.908982
7	1	0	0.116551	1.659201	1.019877
8	1	0	-1.152159	0.888382	-1.263850
9	1	0	0.586412	-2.471444	0.405574
10	1	0	1.542824	-1.55299	-0.773485
11	7	0	-0.522537	-1.039930	-0.709228
12	1	0	-0.741781	-1.387075	-1.627712
13	9	0	-1.959249	0.363180	0.506326
14	9	0	1.254383	1.356644	-0.625714

G° (Hartree-Fock) -411.1348173 Imag. Freq. = 0

4

			Coordinates (Angstroms)		
Center Number	Atomic Number	Atomic Type	X	Y	Z
1	6	0	1.444511	0.571046	0.409603
2	6	0	-0.044309	0.863379	0.521698
3	6	0	-0.696723	-0.541759	0.494408
4	6	0	1.474827	-0.666831	-0.503171
5	1	0	1.992709	1.432333	0.025534
6	1	0	1.837875	0.319112	1.399600
7	1	0	2.347158	-1.299338	-0.331547
8	1	0	1.473315	-0.371560	-1.556569
9	1	0	0.237530	-1.383457	-0.163361
10	1	0	0.345511	-2.307763	0.220736
11	7	0	-0.958898	-0.884869	1.500328
12	1	0	-0.335344	1.441402	1.399288
13	9	0	-1.926425	-0.480142	-0.185083
14	9	0	-0.455227	1.591239	-0.598146

G° (Hartree-Fock) -411.1255847 Imag. Freq. = 0

5 = 4

7 = 6 = 2

8 = 1

9

			Coordinates (Angstroms)		
Center Number	Atomic Number	Atomic Type	X	Y	Z
1	6	0	0.763949	-0.421154	0.526499
2	6	0	-0.054227	0.882775	0.521099
3	6	0	-1.485318	0.361275	0.446206
4	6	0	-0.153278	-1.456959	-0.172487

5	1	0	0.996059	-0.709784	1.555074
6	1	0	0.165144	1.550417	1.355388
7	1	0	-1.807701	0.023733	1.439007
8	1	0	-2.183412	1.124054	0.101481
9	1	0	-0.333448	-2.305743	0.493151
10	1	0	0.322464	-1.832502	-1.078678
11	7	0	-1.432071	-0.784667	-0.471384
12	1	0	-1.412534	-0.43146	-1.422812
13	9	0	0.222544	1.588684	-0.657845
14	9	0	1.983141	-0.268571	-0.127802

G° (Hartree-Fock) -411.1169296 Imag. Freq. = 0

10

			Coordinates (Angstroms)		
Center Number	Atomic Number	Atomic Type	X	Y	Z
1	6	0	-0.840288	-0.340015	-0.395815
2	6	0	0.415178	-0.824704	0.334949
3	6	0	0.975938	0.448914	0.956112
4	6	0	-0.604264	1.174517	-0.610022
5	1	0	0.432439	0.661532	1.883399
6	1	0	2.038419	0.372512	1.187173
7	1	0	-1.399127	1.708734	-0.081490
8	1	0	-0.642739	1.461769	-1.660895
9	1	0	0.707780	1.506337	-0.026133
10	1	0	1.418694	1.471844	-0.748083
11	7	0	0.216458	-1.649317	1.019162
12	1	0	-1.018347	-0.909730	-1.307535
13	9	0	1.321722	-1.286673	-0.643758
14	9	0	-1.952793	-0.537102	0.441520

G° (Hartree-Fock) -411.1222198 Imag. Freq. = 0

11 = 10

12

			Coordinates (Angstroms)		
Center Number	Atomic Number	Atomic Type	X	Y	Z
1	6	0	0.078071	0.877514	0.463337
2	6	0	0.619429	-0.555114	0.458574
3	6	0	-0.319531	-1.267174	-0.516695
4	6	0	-1.433744	0.680223	0.262182
5	1	0	0.350526	1.450533	1.349900
6	1	0	0.522279	-0.980233	1.463957
7	1	0	-0.038796	-1.006705	-1.541614
8	1	0	-0.307408	-2.352403	-0.418811
9	1	0	-1.769262	1.387959	-0.500235
10	1	0	-1.995938	0.880384	1.176622

11	7	0	-1.641113	-0.719135	-0.187247
12	1	0	-2.066841	-1.263296	0.552682
13	9	0	0.610067	1.565161	-0.636520
14	9	0	1.959698	-0.620130	0.105837

G° (Hartree-Fock) -411.1160778 Imag. Freq. = 0

13 = 12

15 = 14 = 10

16

			Coordinates (Angstroms)		
Center Number	Atomic Number	Atomic Type	X	Y	Z
1	6	0	0.763141	-0.423859	0.527318
2	6	0	-0.052702	0.882419	0.522735
3	6	0	-1.485217	0.365365	0.441240
4	6	0	-0.158922	-1.458857	-0.166787
5	1	0	0.999228	-0.711842	1.555181
6	1	0	0.165234	1.546701	1.360164
7	1	0	-1.813196	0.028347	1.432396
8	1	0	-2.179203	1.130612	0.093599
9	1	0	-0.348223	-2.299973	0.506168
10	1	0	0.317212	-1.846603	-1.067619
11	7	0	-1.431657	-0.780611	-0.476107
12	1	0	-1.403369	-0.430481	-1.427497
13	9	0	1.979500	-0.274030	-0.132595
14	9	0	0.230067	1.591486	-0.652592

17

			Coordinates (Angstroms)		
Center Number	Atomic Number	Atomic Type	X	Y	Z
1	6	0	-1.179704	0.089144	0.380986
2	6	0	-0.042865	-0.723475	1.015715
3	6	0	1.220210	-0.170727	0.370274
4	6	0	-0.498906	1.300856	-0.282570
5	1	0	-1.948303	0.372320	1.100119
6	1	0	-0.168854	-1.789908	0.835724
7	1	0	-0.005527	-0.547304	2.092791
8	1	0	2.139748	-0.292760	0.942088
9	1	0	-0.914530	2.251824	0.056290
10	1	0	-0.645103	1.217510	-1.364716
11	7	0	0.907363	1.182946	0.128512
12	1	0	1.577959	1.687664	-0.435493
13	9	0	1.463406	-0.899219	-0.849324
14	9	0	-1.838889	-0.673531	-0.598768

G° (Hartree-Fock) -411.1364769 Imag. Freq. = 0

18

Center Number	Atomic Number	Atomic Type	Coordinates (Angstroms) X	Y	Z
1	6	0	-1.187090	-0.281477	0.502085
2	6	0	0.076096	-1.104002	0.295394
3	6	0	1.033177	-0.163620	-0.462511
4	6	0	-0.671042	1.152822	0.539521
5	1	0	-1.780541	-0.570571	1.370525
6	1	0	0.525570	-1.355222	1.259049
7	1	0	-0.383763	1.412593	1.565141
8	1	0	-1.425027	1.865870	0.205536
9	7	0	0.498039	1.148495	-0.344350
10	1	0	0.377831	1.635953	-1.217570
11	1	0	-0.126980	-2.029716	-0.241642
12	9	0	-2.025297	-0.435438	-0.627316
13	1	0	1.213293	-0.444720	-1.500435
14	9	0	2.314907	-0.250782	0.152084

G° (Hartree-Fock) 411.1348201 Imag. Freq. = 0

19

Center Number	Atomic Number	Atomic Type	Coordinates (Angstroms) X	Y	Z
1	6	0	1.085146	-0.218668	0.508728
2	6	0	0.025619	-1.164342	-0.083008
3	6	0	-1.113250	-0.250310	-0.531300
4	6	0	0.461688	1.174921	0.433675
5	1	0	0.461645	-1.690899	-0.932990
6	1	0	1.211548	1.926551	0.185114
7	1	0	0.006050	1.428662	1.399752
8	1	0	-0.526708	1.024724	-0.639318
9	7	0	-1.175948	1.789660	-0.766192
10	1	0	-0.337655	-1.899077	0.634272
11	1	0	-1.642068	-0.553151	-1.433656
12	1	0	1.401115	-0.486769	1.516404
13	9	0	-2.135795	-0.284587	0.498183
14	9	0	2.247690	-0.264041	-0.286632

G° (Hartree-Fock) -411.137674 Imag. Freq. = 0

24 = 21 = 20 = 17

22 =19

23 = 18

Standard orientations for the difluorinated pyrrolidines (DMSO, B3LYP-GD3BJ/6-311++G**)

1

			Coordinates (Angstroms)		
Center Number	Atomic Number	Atomic Type	X	Y	Z
1	6	0	0.282718	-1.332334	0.403692
2	6	0	-0.649786	-0.533484	-0.504240
3	6	0	-0.039251	0.864359	-0.497135
4	6	0	1.648982	-0.631618	0.237427
5	1	0	-0.075348	-1.243315	1.431120
6	1	0	0.315004	-2.387266	0.136172
7	1	0	-0.680697	-0.910156	-1.527087
8	1	0	-0.375409	1.557836	-1.263416
9	1	0	2.346090	-1.234302	-0.348010
10	1	0	2.111105	-0.415685	1.204200
11	7	0	1.314970	0.601491	-0.501392
12	9	0	-0.496441	1.521269	0.762223
13	9	0	-1.977150	-0.538779	-0.037826
14	1	0	1.960803	1.378505	-0.481275

G° (Hartree-Fock) -411.1473709 Imag. Freq. = 0

2

			Coordinates (Angstroms)		
Center Number	Atomic Number	Atomic Type	X	Y	Z
1	6	0	0.746059	0.792271	0.919170
2	6	0	0.673446	-0.628175	0.374730
3	6	0	-0.619595	-0.634673	-0.428434
4	6	0	-0.099783	1.644345	-0.055003
5	1	0	0.315885	0.806386	1.921622
6	1	0	1.781451	1.124975	0.991983
7	1	0	0.730990	-1.417355	1.119773
8	1	0	-0.754471	-1.438120	-1.147181
9	1	0	0.508749	2.336325	-0.640719
10	1	0	-0.855143	2.223530	0.482566
11	7	0	-0.706391	0.639319	-0.942499
12	1	0	-1.504697	0.891067	-1.508843
13	9	0	-1.683151	-0.923445	0.597129
14	9	0	1.741063	-0.859294	-0.539850

G° (Hartree-Fock) -411.147371 Imag. Freq. = 0

3

			Coordinates (Angstroms)		
Center Number	Atomic Number	Atomic Type	X	Y	Z
1	6	0	-0.975470	0.400275	0.980594
2	6	0	-0.365537	-0.837278	0.351886

3	6	0	0.831638	-0.290159	-0.420561
4	6	0	-0.703134	1.501069	-0.055127
5	1	0	-2.033514	0.266257	1.204868
6	1	0	-0.447285	0.606901	1.914749
7	1	0	-0.126851	-1.652147	1.030487
8	1	0	1.148753	-0.891982	-1.268908
9	1	0	-0.561261	2.477014	0.412568
10	1	0	-1.521696	1.580504	-0.776788
11	7	0	0.531679	1.037171	-0.715572
12	1	0	0.707971	1.351236	-1.658458
13	9	0	1.975134	-0.373731	0.511074
14	9	0	-1.265451	-1.364205	-0.621104

G° (Hartree-Fock) -411.1490084 Imag. Freq. = 0

4

			Coordinates (Angstroms)		
Center Number	Atomic Number	Atomic Type	X	Y	Z
1	6	0	-0.282702	-1.332353	0.403666
2	6	0	0.649805	-0.533474	-0.504241
3	6	0	0.039241	0.864357	-0.497139
4	6	0	-1.648965	-0.631622	0.237451
5	1	0	-0.314999	-2.387273	0.136096
6	1	0	0.075376	-1.243392	1.431095
7	1	0	0.680736	-0.910136	-1.527092
8	1	0	0.375404	1.557839	-1.263415
9	1	0	-2.111030	-0.415653	1.204244
10	1	0	-2.346116	-1.234309	-0.347931
11	7	0	-1.314974	0.601465	-0.501419
12	1	0	-1.960811	1.378475	-0.481272
13	9	0	0.496401	1.521272	0.762228
14	9	0	1.977153	-0.538745	-0.037807

G° (Hartree-Fock) -411.1473709 Imag. Freq. = 0

5

			Coordinates (Angstroms)		
Center Number	Atomic Number	Atomic Type	X	Y	Z
1	6	0	-0.479401	-1.269665	-0.459934
2	6	0	0.528000	-0.617270	0.474690
3	6	0	0.135464	0.859079	0.460259
4	6	0	-1.753747	-0.482228	-0.134015
5	1	0	-0.171275	-1.102791	-1.495137
6	1	0	-0.587100	-2.339315	-0.286364
7	1	0	0.437560	-0.992237	1.496355
8	1	0	0.451573	1.443304	1.320832
9	1	0	-2.283364	-0.933885	0.710757
10	1	0	-2.439543	-0.425849	-0.981129

11	7	0	-1.234742	0.852959	0.224955
12	9	0	0.857640	1.498367	-0.657577
13	9	0	1.861067	-0.833764	0.082934
14	1	0	-1.814920	1.459143	0.785782

G° (Hartree-Fock) -411.1469837 Imag. Freq. = 0

6

			Coordinates (Angstroms)		
Center Number	Atomic Number	Atomic Type	X	Y	Z
1	6	0	0.975750	0.400246	0.980413
2	6	0	0.365348	-0.837179	0.351913
3	6	0	-0.831436	-0.289954	-0.420911
4	6	0	0.703454	1.501166	-0.055141
5	1	0	0.447903	0.607016	1.914727
6	1	0	2.033800	0.265882	1.204489
7	1	0	1.522078	1.580884	-0.776713
8	1	0	0.561217	2.477049	0.412587
9	7	0	-0.531009	1.036842	-0.715657
10	1	0	-1.149152	-0.891994	-1.268862
11	1	0	0.126395	-1.651908	1.030559
12	9	0	-1.975636	-0.373507	0.511209
13	9	0	1.265285	-1.364732	-0.620973
14	1	0	-0.710709	1.353657	-1.656951

G° (Hartree-Fock) -411.14903 Imag. Freq. = 0

7 = 2

8 = 4

9

			Coordinates (Angstroms)		
Center Number	Atomic Number	Atomic Type	X	Y	Z
1	6	0	0.717111	-0.491135	0.548382
2	6	0	-0.011113	0.870935	0.554160
3	6	0	-1.469058	0.481567	0.353148
4	6	0	-0.291226	-1.474940	-0.080939
5	1	0	1.013683	-0.778770	1.556374
6	1	0	0.194982	1.482089	1.430778
7	1	0	-1.892188	0.189435	1.320431
8	1	0	-2.061518	1.301029	-0.054041
9	1	0	-0.622391	-2.179391	0.686614
10	1	0	0.160499	-2.040214	-0.896449
11	7	0	-1.447673	-0.685145	-0.544882
12	1	0	-1.265093	-0.358258	-1.490113
13	9	0	0.416440	1.621317	-0.572030
14	9	0	1.909276	-0.414480	-0.204406

G° (Hartree-Fock)	-411.1318907		Imag. Freq. =	0

10

Center Number	Atomic Number	Atomic Type	Coordinates (Angstroms) X	Y	Z
1	6	0	-0.414195	-0.806645	0.347891
2	6	0	0.836325	-0.313158	-0.385499
3	6	0	0.628874	1.207471	-0.550677
4	6	0	-1.033214	0.461674	0.913877
5	1	0	-0.208615	-1.599483	1.064979
6	1	0	1.372906	1.714332	0.071324
7	1	0	0.755102	1.537705	-1.580947
8	1	0	-0.539060	0.690645	1.864165
9	1	0	-2.104588	0.367888	1.091239
10	1	0	-0.729623	1.516865	-0.066192
11	7	0	-1.380739	1.434300	-0.841756
12	1	0	-1.293903	-1.350088	-0.633091
13	9	0	0.998851	-0.853542	-1.316552
14	9	0	1.972432	-0.561684	0.428351

G° (Hartree-Fock)	-411.1351838		Imag. Freq. =	0

11 = 10

12

Center Number	Atomic Number	Atomic Type	Coordinates (Angstroms) X	Y	Z
1	6	0	0.083915	0.869491	0.475331
2	6	0	0.592211	-0.570169	0.464910
3	6	0	-0.346687	-1.267099	-0.516528
4	6	0	-1.422155	0.712156	0.242243
5	1	0	0.357816	1.438740	1.360869
6	1	0	0.501017	-0.996238	1.466430
7	1	0	-0.058665	-1.015068	-1.542298
8	1	0	-0.357360	-2.350740	-0.407671
9	1	0	-1.726459	1.419284	-0.533927
10	1	0	-1.992050	0.932754	1.145279
11	7	0	-1.661184	-0.692068	-0.189989
12	1	0	-2.050516	-1.207748	0.592280
13	9	0	0.664181	1.557089	-0.627902
14	9	0	1.948130	-0.650731	0.100482

G° (Hartree-Fock)	-411.132017		Imag. Freq. =	0

13 = 12

15 = 14 = 10

16 = 9

17

			Coordinates (Angstroms)		
Center Number	Atomic Number	Atomic Type	X	Y	Z
1	6	0	-1.187346	0.050100	0.375980
2	6	0	-0.009639	-0.546091	1.141034
3	6	0	1.201682	-0.032767	0.384338
4	6	0	-0.630222	1.298827	-0.314085
5	1	0	-2.063540	0.242746	0.990380
6	1	0	-0.055262	-1.632341	1.201924
7	1	0	0.008950	-0.139668	2.155266
8	1	0	2.156461	-0.047984	0.903536
9	1	0	-1.041905	2.210577	0.124451
10	1	0	-0.889950	1.278312	-1.376214
11	7	0	0.819170	1.222580	-0.072998
12	1	0	1.436854	1.704677	-0.711999
13	9	0	1.451503	-0.985855	-0.747420
14	9	0	-1.621797	-0.880233	-0.619243
G° (Hartree-Fock)	-411.15235		Imag. Freq. =	0	

18

			Coordinates (Angstroms)		
Center Number	Atomic Number	Atomic Type	X	Y	Z
1	6	0	-1.181672	-0.277894	0.512496
2	6	0	0.074072	-1.103632	0.292484
3	6	0	1.017656	-0.164976	-0.472710
4	6	0	-0.668733	1.153234	0.542545
5	1	0	-1.775113	-0.567526	1.377825
6	1	0	0.515674	-1.351083	1.260881
7	1	0	-0.379200	1.404306	1.568012
8	1	0	-1.415801	1.872888	0.208851
9	7	0	0.503925	1.148697	-0.348858
10	1	0	0.351149	1.607709	-1.236012
11	1	0	-0.126226	-2.031979	-0.241226
12	9	0	-2.039462	-0.434351	-0.625392
13	1	0	1.216314	-0.443875	-1.504994
14	9	0	2.332549	-0.251394	0.154256
G° (Hartree-Fock)	-411.1509097		Imag. Freq. =	0	

19

			Coordinates (Angstroms)		
Center Number	Atomic Number	Atomic Type	X	Y	Z
1	6		1.094192	-0.227227	0.529331
2	6		0.013348	-1.167025	-0.018407

3	6	-1.077570	-0.242105	-0.552063
4	6	0.476731	1.162274	0.472360
5	1	0.426144	-1.786094	-0.816226
6	1	1.219258	1.931409	0.259130
7	1	-0.000942	1.389008	1.433384
8	7	-0.488842	1.022317	-0.625068
9	1	-1.112395	1.801227	-0.798566
10	1	-0.384681	-1.826244	0.752216
11	1	-1.582725	-0.548675	-1.464042
12	1	1.472625	-0.498838	1.511746
13	9	-2.189306	-0.261671	0.444517
14	9	2.227573	-0.268720	-0.343349

G° (Hartree-Fock) -411.1525187 Imag. Freq. = 0

24 = 21 = 20 = 17

22 =19

23 = 18
